# Physicochemical and sensory characteristics of fermented sheepmeat sausage

**DOI:** 10.1002/fsn3.151

**Published:** 2014-07-23

**Authors:** Yanjun Lu, Owen A Young, John D Brooks

**Affiliations:** School of Applied Sciences, Auckland University of TechnologyAuckland, New Zealand

**Keywords:** 4-methyloctanoic acid, fermentation, sheepmeat, skatole, spicing

## Abstract

The aim of the study was to compare the physicochemical and sensory characteristics of fermented, cured sausages made from equivalent muscle groups of beef, pork, and sheepmeat. The last has no commercial examples and represents an unexploited opportunity. Using seven replicates of shoulder meat and subcutaneous fat, sausages were made with 64%, 29%, 4%, 2%, 0.2%, and 0.01% of lean meat, fat, NaCl, glucose, sodium pyrophosphate, and lactic culture, respectively. Following anaerobic fermentation (96 h, 30°C), there were no significant differences between the species in mean texture (hardness, springiness, adhesiveness, cohesiveness) and pH, and only minor differences were seen in color. However, although not consumer tested, it is argued that consumers would be able to pick a texture difference due to different fat melting point ranges, highest for sheepmeat. This work was followed by a sensory experiment to find out if characteristic sheepmeat flavors could be suppressed to appeal to unhabituated consumers. To simulate a very strongly characteristic sheepmeat, beef sausage mixtures (above) were spiked, or not, with 4-methyloctanoic, 4-methylnonanoic acid, and skatole (5.0, 0.35, and 0.08 mg kg^−1^, respectively). Sodium nitrite (at 0.1 g kg^−1^) and a garlic/rosemary flavor were variably added to create a 2^3^ factorial design. In a randomized design, 60 consumers found that spiked sheepmeat flavors caused an overall significant decrease in mean liking on a 1–9 scale (5.83 vs. 5.35,*P* = 0.003), but this was completely negated by the garlic/rosemary addition (5.18 vs. 6.00,*P* < 0.001). Nitrite had no effect on liking (5.61 vs. 5.58,*P* = 0.82), although nitrite might be included in commercial examples to minimize fat oxidation and suppress growth of clostridia. Thus, sheepmeat flavors could be suppressed to appeal to unhabituated consumers. Commercial examples could thus be made for these consumers, but the mandatory use of the name “mutton” in some markets would adversely affect prospects.

## Introduction

Worldwide, cured fermented sausages are almost all made from beef and pork, with differences stemming from species, different genera of lactic acid bacteria, fat-to-lean ratio, salt and sugar content, the use of spices based on cultural familiarity, and the degree of drying. Fermented sausages made from other animal meats are rarer, but the production principles remain the same; in the presence of salt and sugars, and as pH falls as lactic acid accumulates, myosin forms a gel that binds the meat particles forming a preserved, sliceable mass, usually in the form of sausage (Cocolin and Rantsiou 2012).

Possibly because of sheep's hardiness to cold winters, sheepmeat is seldom preserved by fermentation and there are no clear examples of commercial fermented sheepmeat sausages. There is no technical reason why fermented sheepmeat sausages could not be developed, but the physical properties of such a product might differ substantially from that of beef and pork equivalents, and there may be flavor problems as discussed below. Thus, the first and shorter part of this research compares the physicochemical properties of fermented sausages prepared from beef, pork, and sheepmeat. Fermented sausages are usually prepared from cheaper meat cuts, which have higher concentrations of collagen, because comminution overcomes toughness problems. Shoulder meat was chosen for this work, having a higher concentration of collagen than hindquarter meat (Casey et al. 1986). To minimize within-species variability due to exact cut and animal effects, multiple purchases were made from retail butchers. Subcutaneous carcass fat of each species was obtained at the same time, but information on the exact source site on the carcass was unavailable. Fermented sausages were made to a basic formula and compared for growth of lactic acid bacteria, final pH, color (CIE L*, a*, b*), and texture (hardness, springiness, cohesiveness, adhesiveness). The hypothesis was that sausages from the three species would be significantly different for many of these properties.

Flavor is another issue. Sheepmeat (and goat meat) has a characteristic flavor caused by trace concentrations of branched chain fatty acids (BCFAs) (Wong et al. 1975). The BCFAs are typified by 4-methyloctanoic acid (4-MeO) and 4-methylnonanoic acid (4-MeN), which like the dominating fatty acids—stearic and oleic, etc.—are esterified in body fats, but released to an extent as free acids on cooking and thus contributing to flavor. Appreciation of this flavor is an acquired taste, and is not generally liked in many parts of the United States, and in Japan, for example (Prescott et al. 2001). A second flavor of concern is due to skatole (3-methylindole) a fecal-smelling decarboxylation product of dietary tryptophan (Yokoyama and Carlson 1974). It can accumulate in body and milk fat when animals are raised on pasture as is the norm in Australasia (Young et al. 1997, 2003). Skatole flavor is disliked in high concentrations (Prescott et al. 2001).

The hypothesis tested here was that nitrite curing and/or spicing of salted, fermented sheepmeat sausage may be able to suppress, by whatever sensory mechanism, either or both flavors. The sausage style chosen for research was undried and unsmoked, the reason being that if a basic sausage model from sheepmeat were acceptable to consumers, then derived products would also be likely to be acceptable. Garlic is the spice most widely used with sheepmeat throughout the world (Smith and Young 1993) and rosemary is also typically used in Western cuisine. In combination, these two spices were chosen to represent the spicing variable in the experiment (Spice or No spice). The curing variable was nitrite added (Cure) or not added (No cure).

In assessing the ability of these experimental conditions to suppress species- and diet-based flavors, it was important to have a no-sheepmeat control, ideally a sheepmeat with low to zero BCFAs and skatole concentrations. No such sheepmeat has been identified. Rather, we adopted the strategy of Prescott et al. (2001) who tested the relative acceptance of sheepmeat by people habituated (New Zealand) and unhabituated (Japan) to sheepmeat. They did this by adding quantities of BCFAs and skatole to grain-finished ground beef, the meat known to have negligible concentrations of both. Thus, they generated a synthetic sheepmeat. Grain-finished beef was unavailable for the present work so pasture-finished beef was used instead, while realizing that a pastoral diet is likely to generate some skatole in all ruminants.

## Material and Methods

### Chemicals

Salt, glucose, Na_4_P_2_O_7_·10H_2_O, and sodium nitrite were sourced from a variety of suppliers. A fermentation culture mixture (*Pediococcus pentosaceus* and*Staphylococcus carnosus*, BFL-F02 BactoFlavor®) was donated by Chr. Hansen, Melbourne, Australia. Rosemary essential oil (FN11146) and garlic essential oil (FN11516) were from Lionel Hitchen (Essential Oils) Limited (Winchester, UK). The BCAFs, 4-MeO and 4-MeN, and skatole were from Sigma, Sydney, Australia.

### Meat source and sausage preparation for the physicochemical study

Shoulder meat and subcutaneous fat of the three species were each bought from seven different retailers on seven occasions over a period of months. On the day of each purchase, the meat of each species was trimmed of visible fat and diced into cubes about 15 mm on edge. The fat was similarly trimmed of lean and diced. Leans and fats were individually vacuum packed and held frozen at −80°C for up to a week. After warming to the edge of thawing, meat and fat were separately ground through a domestic food processor (Kenwood KM300, Kenwood, Havant, UK) fitted with chilled grinder attachment and a 4 mm plate. Ground lean and fat and other ingredients (Table1) were then blended for 3 min in the mixer bowl, then finally reground and extruded into modified 50 mL syringe barrels (BD Plastipak, 300865, Becton Dickinson, Drogheda, Ireland); the entire end disk and Luer lock had been excised by lathe creating an open-ended cylinder (25 mm internal diameter) that was internally lubricated with petroleum jelly. The filled barrels (three for each species on each of 7 days) were sealed with plastic film then aluminum foil to exclude air, and the prepared assemblies were incubated at 30°C for 96 h (called Day 4) (Khem et al. 2013). Cylindrical disks of fermenting sausage were extruded daily to monitor physicochemical properties. Barrel ends were resealed after each extrusion.

**Table 1:** Formula of the sausage meat mixtures for the two experiments.

Ingredient	Final concentrations (g kg^−1^)^1^	
	Physiochemical	Sensory
	mandatory	mandatory
Lean shoulder meat	639	639
Subcutaneous fat	299	299
NaCl	39.9	39.9
Glucose	20.0	20.0
Na_4_P_2_O_7_·10H_2_O	2.0	2.0
Fermentation culture	0.1	0.1
		Variably added
Sodium nitrite^2^	0.1	0.1
Rosemary + garlic extracts^3^		40.0 + 40.0 (mg kg^−1^)
4-MeO + 4-MeN + skatole		5.0 + 0.35 + 0.08 (mg kg^−1^)

1 Less than kilogram quantities were made for the physicochemical study, but data are expressed per kg for clarity.

2 Unlike commercial practice, sodium nitrite (as required) and NaCl were added separately.

3 Mixed with NaCl before addition as required.

### Physicochemical analyses

To measure pH, a 5-g portion from each sample was dispersed into 50 mL of deionized water (Honikel 1998), and the pH was measured by a Meterlab PHM201 from Radiometer, Crawley, UK, fitted with a conventional glass electrode.

Texture profile analysis (Bourne 1978) was performed on Days 0 and 4 on extruded cylinders of fermented sausage with a TAXT Texture Analyser (Stable Microsystems, Godalming, UK) according to Khem et al. (2013). In outline, 30-mm high samples were extruded from the three syringes per species on Days 0 and 4. Each cylinder was compressively probed by a flat aluminum surface, 50 mm in diameter attached to the analyzer's mobile shaft. Important variables were two compressions with a probe speed of 5 mm sec^−1^ to 50% strain. Hardness, springiness, adhesiveness, and cohesiveness were calculated according to Bourne (1978).

Color was measured by a reflectance spectrophotometer (Model 45/0; Hunterlab ColorFlex, Reston, VA). A 5-mm high × 25-mm diameter sausage disk was extruded from each of the nine syringes (three from each of beef, pork, sheepmeat) and placed in the center of a glass crystallizing dish placed over the light exposure cavity. Triplicate L*, a*, and b* data from each disk were each corrected for the mean color of the empty dish.

### Meat source and sausage preparation for the sensory study

**Figure 1:**
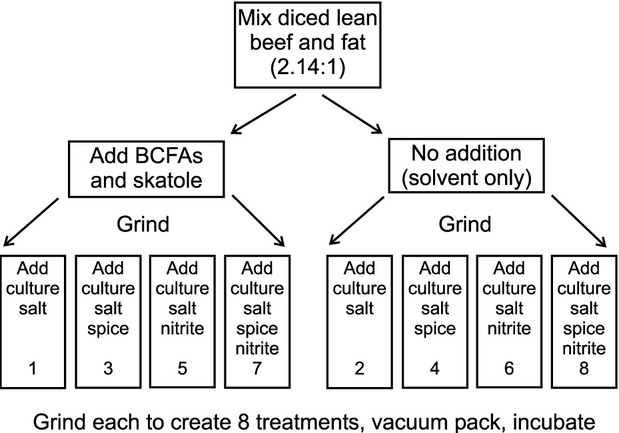
Production flow chart for the eight treatments.

In this experiment, eight treatments were required to satisfy the 2^3^ design. To achieve this, lean beef and fat bought at retail were diced and hand mixed in the ratio of 2.14:1 (lean:fat), sufficient for the entire experiment (Fig.1). One half was to become the BCFAs/skatole treatment and the other was the No-BCFAs/skatole treatment. Solutions of the BCFAs and skatole were prepared in ethanol, and addition to the fat was achieved with a 10 *μ*L glass syringe, whereby minute aliquots were injected into the fat pieces with the aim of achieving an even distribution after grinding. In the No-BCFAs/skatole treatment half an equal volume of ethanol was similarly added to the diced fat (Fig.1). Based on Prescott et al. (2001), the final concentrations of 4-MeO, 4-MeN, and skatole in four of the final eight treatments were 5, 0.35, and 0.08 mg kg^−1^, respectively (Table1). After further hand mixing of the two lean and fat lots, they were ground once through a 4-mm plate of the grinder.

The two ground lots were each divided into four equal sub lots. The other ingredients were then added as required (Fig.1). Based on the manufacturer's specification, the garlic and rosemary extracts were added to the salt before mixing to achieve a final concentration of 40 mg kg^−1^ for each (Table1). However, nitrite was added separately from salt in the four treatments where it was required. Each of the eight treatments was extensively hand mixed to distribute the ingredients evenly, then reground through the 4 mm plate, and packed into flat 1 L plastic food storage containers. These were lightly closed and vacuum packed in barrier bags, an action that also served to seal the lids. Incubation was at 30°C for 96 h (Day 4), with subsequent refrigeration until needed for microbiological assay and sensory evaluation.

### Media and microbiology

Microbiological media were bought from Fort Richard Laboratories, Auckland, New Zealand, to monitor fermentation and to confirm that the panelists were to consume a safe product. The media were de Man–Rogosa–Sharpe (MRS), peptone water, Baird–Parker, MacConkey broth base, eosin methylene blue, tryptone water, and brilliant green bile broth.

The growth of lactic acid bacteria was determined by conventional methods stemming from De Man et al. (1960). After incubation in a CO_2_ atmosphere at 30°C for 48 h, bacteria were determined as colony-forming units per gram. Because the treatment preparation required extensive handling, treatments were tested for consumer safety; tests for*Staphylococcus aureus*, total coliforms, and fecal coliforms followed conventional microbiological methods (Cochran 1950).

### Sensory evaluation

The treatments were evaluated by consumers drawn from the staff members and students ranging from 18 years to 65 years and older. Many of the consumers were young Chinese from many provinces of China, and most—if not all—would have experienced sheepmeat flavor knowingly or unknowingly as a food ingredient in New Zealand. Staff consumers would all have consumed sheepmeat.

Evaluation was done in four booths under red lights because half the treatments were red due to nitrite curing. To eliminate order effects, a fully balanced randomized design was used (Macfie et al. 1989) such that the eight treatment samples—each assigned a random three-digit blinding code—were equally presented in every tasting order. This required eight tasting orders, enough for 64 consumers. In the event there were only 60 consumers, but the small order imbalance was ignored in data analysis.

The sausage treatments were cut into a 25 × 40 × 5 mm slices. Two samples presented within each bay were placed in the required left-to-right tasting order on a flat white foam tray, above their three-digit codes. Prepared trays were kept at 4°C prior to tasting, but warmed quickly because the slices were thin. Consumers were required to eat small apple slices and sip water within and between bays.

Consumers scored only for liking of flavor. This was done on a vertical 9-point category scale ranging 1 (dislike extremely) to 9 (like extremely). Gender and age category data were also collected, the latter in two groups, 18 years to 30, and older to 65. An immediate reward for participation was a chocolate bar and a later chance to win a $50 cash prize by ballot.

### Data analysis

Raw physicochemical data from the three syringes per species were first averaged, creating seven replicates per species given there were seven preparation days. These data were variously analyzed for variance by the*t*-test and analysis of variance (ANOVA) routines in XLSTAT (Addinsoft, New York, NY) within Microsoft Excel. In the sensory experiment, BCFAs/skatole, Spice, and Cure were fixed treatment effects, while consumer, gender, and age group were random effects.

## Results

### Physicochemical experiment

The initial mean pH values of the sausage mixtures were between 5.5 and 6 with no significant differences between the three species (data not shown). At each subsequent day there were similarly no significant differences between species as mean pH values fell to around 4.6 (Table2). The pH fall reflected the growth of lactic acid bacteria, from around an initial log 7.5 to around a final 9.3 colony-forming units per gram, with no significant differences between species at Day 4 (96 h) (Table2) or on previous days (data not shown). Similarly, the theme was continued with texture analysis with no significant differences between species, although as expected most of the properties changed between Days 0 and 4; for example, mean hardness increased sixfold after gelation (data not shown). However, although the texture differences at Day 4 were not statistically significant, means of sheepmeat and beef were consistently closer to each other than means of sheepmeat and pork, or means of beef and pork (Table2).

**Table 2:** Results of the physicochemical comparison after fermentation was completed at Day 4 (96 h).

Attribute	Species		
	Beef	Pork	Sheepmeat
pH	4.63 ± 0.48	4.51 ± 0.46	4.61 ± 0.37
Colony-forming units g^−1^	9.25 ± 0.08	9.27 ± 0.03	9.42 ± 0.03
Hardness (N)	21.38 ± 5.68	23.69 ± 5.12	23.22 ± 6.76
Springiness	0.880 ± 0.071	0.870 ± 0.087	0.881 ± 0.086
Cohesiveness	0.645 ± 0.071	0.742 ± 0.046	0.647 ± 0.047
Adhesiveness (N.sec)	−42.2 ± −35.7	−15.7 ± −6.31	−44.2 ± −31.9
Color			
L^*^	20.8 ± 7.2	21.6 ± 6.7	17.8 ± 7.4
a^*^	14.2 ± 1.6	13.9 ± 2.0	15.0 ± 2.2
b^*^	15.2 ± 1.7^a^	11.6 ± 2.1^b^	11.5 ± 3.6^b^

Data are means ± standard deviations. No superscripts means the differences between species were not significant, while different superscripts means significantly different at*P* < 0.01.

Color was the only variable where a significant difference was observed between species, for b*, but not L* nor a*. The b* (yellowness/greenness) value for beef was numerically higher than for pork and sheepmeat on Days 1 to 4, but significantly so only on Day 4 (Table2). The color difference probably arose from the tendency of bovines to accumulate*β*-carotene from pasture in their body fat (Yang et al. 1992).

It was concluded that within the limits of the lean meat and fat selection method on seven occasions over several months, there were no physicochemical differences in interest between the fermented sausages made from the three species. This disproved the hypothesis that sausages from the three species would be significantly different for the parameters measured. Sheepmeat was as functional as the other two species. Attention was next directed to sheepmeat flavor by a sensory experiment.

### Sensory experiment

No gas was generated in the vacuum packs in the 96 h incubation and the taste of the fermented sausage was tart, which is a characteristic of successful lactic acid fermentation. Hygiene safety was confirmed by assays for*S. aureus* (negative), fecal coliforms (negative), and total coliforms (the most probable number [Cochran 1950] was <23 colonies g^−1^).

Of the 60 consumers, 37 were 30 years or under and mostly young Chinese students, while 23 were older and would have had much greater exposure to sheepment. In spite of this difference, there was no effect of age group on liking (*P* = 0.25) and, therefore, no significant interactions between age and gender or between age and the eight treatments (all*P*s >0.92). Age was subsequently ignored as a factor.

Gender had a significant effect on liking (*P* = 0.001), where the mean liking by the 40 males, 5.78 ± 1.75, was greater than that for the 20 females, 5.21 ± 1.95. However, the gender by treatment interaction was insignificant (*P* = 0.87), so gender as a factor was also ignored.

**Figure 2:**
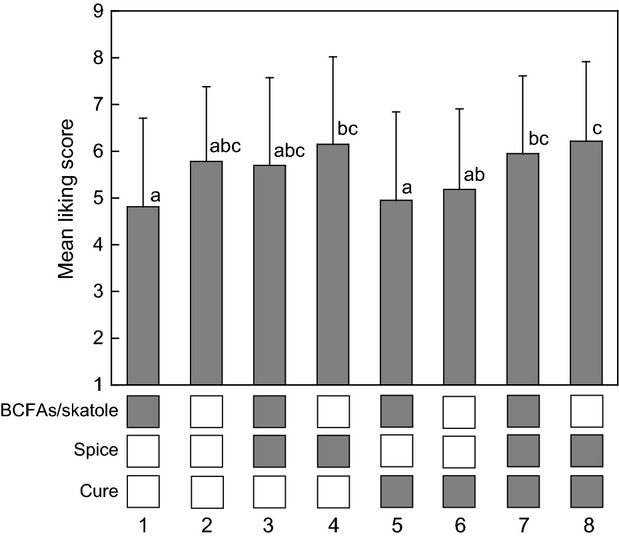
Effect of BCFAs/skatole, spice, and cure combinations on the mean liking of fermented sausage. Shaded squares in the lower section mean that factor was applied to that treatment; numbers 1–8 are referred to in the text. Vertical lines are standard deviations and means with different letters are significantly different at*P* < 0.05.

Figure2 (upper) shows the means and standard deviations for liking of the eight treatments for which*P* < 0.001. Inspection suggests that Cure (bars 5–8 vs. 1–4) had no significant effect on liking, and this was confirmed by analysis of variance (*P* = 0.82, Table3). Compared with the no-addition control (bar 2), addition of BCFAs/skatole but no Spice (bars 1 and 5), resulted in the lowest scores (4.82 and 4.95) that were significantly different from three treatments, bars 4, 7, and 8. All these three had spice added. Numerically, the two most favored treatments were 4 and 8 (6.15 and 6.22), due to Spice alone with no effect due to Cure. Analysis of variance showed there were no significant interactions between any combination of BCFAs/skatole, Spice and Cure, so data could be selectively pooled to isolate the effects of these three factors in the context of the entire experiment (Table3). In both absolute numerical and statistical terms, Spice was the most influential in the liking score and was very effective in suppressing the adverse flavors due to BCFAs and/or skatole. The flavor hypothesis was confirmed for Spice, but not for Cure.

**Table 3:** Isolated effects of BCFAs/skatole, Spice, and Cure on mean liking of fermented sausage, and their statistical significance.

Addition	Mean liking score ± standard deviation		*P*-value
	Added	Not added	
BCFAs/skatole	5.35 ± 1.88	5.83 ± 1.76	0.003
Spice	6.00 ± 1.78	5.18 ± 1.81	<0.001
Cure	5.58 ± 1.81	5.61 ± 1.87	0.82

## Discussion

Although the differences between the three species were not statistically significant for any of the texture profile parameters, pork sausage mean values were slightly numerically different from sheepmeat and beef equivalents. If more replicates were analyzed, it is likely that a significant difference would emerge between pork (porcine) compared with sheepmeat and beef (ruminants, ovine, and bovine). The origins of these putative differences between species could derive from the gelation property differences of porcine and ruminant forequarter muscle proteins, and/or from fat composition. There are no known reports comparing low-pH- and high-salt-generated gelation properties of porcine and ruminant forequarter myosins, although it is known that fast-twitch and slow-twitch myosins from within one species (beef) have markedly different heat-mediated gelation properties (Young et al. 1992). Moreover, the chemical—and thus the textural properties—of porcine and ruminant fats are different, but affected to diet (Wood et al. 2004). The melting point and, thus, hardness of fat is closely related to the proportion of unsaturated fat (Enser et al. 1996). Data from Sivakumaran et al. (2012) that approximates the fats used here, report percent unsaturated values of 42%, 45%, and 57%, for sheepmeat, beef, and pork, respectively, where the former two would be pasture-fed and the pork concentrate-fed. Pork fat would have the lowest melting point range. Although the texture analyzer did not detect statistically significant differences between the three species, it is likely that consumers would detect differences, particularly for pork with its lower melting point range. It is well known that cold sheepmeat fat has a tendency to coat the buccal cavity, and how this phenomenon—independent of flavor problems—would be accepted by consumers used to fermented pork sausages remains unknown.

Although texture of fermented sheepmeat sausage might be acceptable to the unhabituated consumer, the flavor may not. Thus, Prescott et al. (2001) showed that Japanese consumers, who are unhabituated to sheepmeat, increasingly disliked the increasing concentrations of BCFAs spiked into grain-finished beef, certainly more than a habituated population of New Zealand residents. Neither population liked high concentrations of spiked skatole. The sensory results reported here show that adverse flavor problems caused by these compounds can be solved by at least the two spices used in combination here. In this respect, other spices remain untested, but it seems likely that other spices commonly used with sheepmeat (Smith and Young 1993) may also be effective in flavor suppression where required.

Although the panel comprised only 40 males and 20 females, there was a marked gender effect for liking these lactic acid-fermented sausage (5.78 vs. 5.21,*P* = 0.001). This result was consistent with results of a goat milk yogurt study where males reliably liked a variety of goat yogurts more than females (Young et al. 2012). (Goat milk also contains BCFAs.) Similarly, Young et al. (2009) showed that females were much better than males at correctly identifying sheepmeat whose flavor had been modified by curing and flavoring with the sugar xylose. It seems highly likely that females are more sensitive to BCFAs than males, as first proposed from perfumery experiments by Boelens et al. (1983).

The current sensory study also showed that curing had no effect on liking of fermented sausage. Arguably nitrite could be omitted from fermented sheepmeat sausage, perhaps a commercial advantage in sophisticated markets where there is a widespread, but misplaced, distrust of additives to foods (Haen 2014). Any real or perceived problems arising from nitrite-derived nitrosamine formation can easily be prevented by ascorbic acid addition (Martin 2012). However, there are three reasons why nitrite should be retained. First, cured color is well accepted in gourmet meats; second, nitrite is a powerful inhibitor of clostridia species (Perigo and Roberts 1968); and third, nitrite is a useful antioxidant, and if the models developed here were to be partially air dried, as is common for fermented sausage, fat oxidation would be minimized (Ladikos and Lougovois 1990).

In developing fermented products from sheepmeat off pasture feeding, tougher and thus cheaper meat from older sheep would be the most likely raw material. For historical/cultural reasons, the legal names in Australasia for sheepmeat of increasing age are lamb, hogget, and mutton. Mutton is tougher than lamb (Young and Braggins 1998), and has higher concentrations of BCFAs (Young et al. 2006). Mutton toughness is overcome by comminution in fermented sausage, and as shown here spicing can suppress BCFA and skatole flavor where this is required for the unhabituated. However, as an ingredient, the meat name, in this case mutton, must be specified on food labels (Food Standards Australia New Zealand 2013). Young and Lim (2001) found that consumer perceptions of “quality,” “flavor,” and “healthiness” on name and name-alone, all markedly declined in the sequence: lamb > hogget > mutton. Thus, although it is clearly possible to make flavor-acceptable fermented sheepmeat sausage, marketing in at least the domestic markets would be difficult because of an historical regulation.

## Conflict of Interest

None declared.
